# A global survey about undiagnosed rare diseases: perspectives, challenges, and solutions

**DOI:** 10.3389/fpubh.2025.1510818

**Published:** 2025-02-26

**Authors:** Simone Baldovino, Savino Sciascia, Claudio Carta, Marco Salvatore, Laura L. Cellai, Gianluca Ferrari, Aimé Lumaka, Stephen Groft, Yasemin Alanay, Maleeha Azam, Gareth Baynam, Helene Cederroth, Eva Maria Cutiongco-de la Paz, Vajira Harshadeva Weerabaddana Dissanayake, Roberto Giugliani, Claudia Gonzaga-Jauregui, Dineshani Hettiarachchi, Oleg Kvlividze, Guida Landoure, Prince Makay, Béla Melegh, Ugur Ozbek, Karaman Pagava, Ratna Dua Puri, Vaness I. Romero, Vinod Scaria, Saumya S. Jamuar, Vorasuk Shotelersuk, Dario Roccatello, William A. Gahl, Samuel A. Wiafe, Olaf Bodamer, Manuel Posada, Domenica Taruscio

**Affiliations:** ^1^Center of Excellence on Nephrologic, Rheumatologic, and Rare Diseases (ERK-Net, ERN-Reconnect, and RITA-ERN Member) with Nephrology and Dialysis Unit, Coordinating Center of Piedmont, Valle d’Aosta Network for Rare Diseases, Department of Clinical and Biological Sciences, San Giovanni Bosco Hub Hospital, University of Turin, Turin, Italy; ^2^National Centre for Rare Diseases, Istituto Superiore di Sanità, Rome, Italy; ^3^National Center Rare Diseases - Undiagnosed Rare Diseases Interdepartmental Unit, Istituto Superiore di Sanità, Rome, Italy; ^4^Reference Center for Rare and Undiagnosed Diseases, University of Kinshasa, Kinshasa, Democratic Republic of Congo; ^5^National Center for Advancing Translational Sciences, National Institutes of Health, Bethesda, MD, United States; ^6^ACURARE-Rare and Undiagnosed Diseases Center, Acibadem University, Istanbul, Türkiye; ^7^Translational Genomics Laboratory, Department of Biosciences, COMSATS University Islamabad, Islamabad, Pakistan; ^8^Rare Care, Clinical Centre of Expertise for Rare and Undiagnosed Diseases, Perth Children's Hospital, Perth, WA, Australia; ^9^Wilhelm Foundation, Stockholm, Sweden; ^10^Institute of Human Genetics, National Institutes of Health, University of the Philippines Manila, Manila, Philippines; ^11^Department of Anatomy, Genetics, and Biomedical Informatics, Faculty of Medicine, University of Colombo, Colombo, Sri Lanka; ^12^House of Rares, Department of Genetics UFRGS and DASA, Medical Genetics Service, HCPA, Porto Alegre, Brazil; ^13^International Laboratory for Human Genome Research, Universidad Nacional Autonoma de Mexico, Queretaro, Mexico; ^14^Georgian Foundation for Genetic and Rare Diseases (GeRaD), School of Medicine, New Vision University, Tbilisi, Georgia; ^15^Faculté de Médecine et d'Odontostomatologie, l'Université des Sciences, des Techniques et des Technologies de Bamako, Bamako, Mali; ^16^Department of Medical Genetics, School of Medicine, University of Pécs, Pécs, Hungary; ^17^Department of Child and Adolescent Medicine, Tbilisi State Medical University, Tbilisi, Georgia; ^18^Institute of Medical Genetics and Genomics, Sir Ganga Ram Hospital, New Delhi, India; ^19^School of Medicine, Universidad San Francisco de Quito, Quito, Ecuador; ^20^CSIR Institute of Genomics and Integrative Biology, New Delhi, India; ^21^Genetics Service, Department of Paediatrics, KK Women’s and Children’s Hospital and Paediatric ACP, Duke-NUS Medical School, and SingHealth Duke-NUS Institute of Precision Medicine, Singapore, Singapore; ^22^Center of Excellence for Medical Genomics, Department of Pediatrics, Faculty of Medicine, King Chulalongkorn Memorial Hospital, Chulalongkorn University, Bangkok, Thailand; ^23^National Institutes of Health, National Human Genome Research Institute, Bethesda, MD, United States; ^24^Rare Disease Ghana Initiative, Accra, Ghana; ^25^Division of Genetics and Genomics, Harvard Medical School, Boston Children's Hospital, Boston, MA, United States; ^26^Rare Diseases Research Institute (IIER), SpainUDP, Instituto de Salud Carlos III (ISCIII), Madrid, Spain

**Keywords:** healthcare disparities, undiagnosed rare diseases (URDs), people living with URDs (PLURDs), diagnostic journey, advocacy groups, genomic diagnosis

## Abstract

**Background:**

Undiagnosed rare diseases (URDs) are a complex and multifaceted challenge, especially in low-and medium-income countries. They affect individuals with unique clinical features and lack a clear diagnostic label. Although the Undiagnosed Diseases Network International (UDNI) definition of URDs is not universally accepted, it is widely recognized.

**Methods:**

We surveyed UDNI members and participants from other countries to explore the challenges posed by URDs and identify possible solutions. Participation in the survey was completely voluntary.

**Results:**

The survey revealed a need for more consensus on a universally accepted definition for URDs. Still, the UDNI definition gained widespread recognition and serves as a valuable framework for understanding and addressing the challenges of URDs. In addition to national or international networks, fostering a more substantial engagement and resource-sharing ethos among member countries is critical. Despite advances in genomics and diagnostic tools, the diagnostic journey for people living with URDs (PLURDs) remains arduous and often inconclusive. The availability of specialized centers and the utilization of whole exome sequencing (WES) and whole genome sequencing (WGS) vary across countries, with disparities due to healthcare systems, economic status, and government policies. Advocacy groups play a crucial role in supporting PLURDs.

**Conclusion:**

A unified commitment to prioritizing URDs on the global health agenda, paired with targeted funding, stipulated national strategies, and aligned international cooperation, is imperative to leveling the playing field for the diagnosis and management of URDs and capitalizing on the potential of Advocacy Groups as allies in this endeavor.

## Introduction

In the past 40 years, there has been increased attention to rare diseases (RDs). This attention was prompted by concerns surrounding the absence of treatment options and other factors, such as diagnostic challenges and the severity of the diseases, including their potential to be life-threatening (1). Currently, most RD definitions rely on epidemiological data, specifically low prevalence rates, even if no universally shared definitions exist. For example, in the European Union, a disease that affects fewer than 5 in 10,000 individuals is classified as rare, while in the United States, a condition that impacts fewer than 200,000 people nationwide is considered rare (2). As reported by Haendel et al. (3), when primary knowledge sources on rare diseases (such as Orphanet, OMIM, GARD, DOID, and NCI Thesaurus) are combined algorithmically and curated within Monarch Disease Ontology, more than 10,000 rare disease “leaf terms” can be identified, and about 200 new rare diseases are described yearly (4). One recent study of the global prevalence of rare diseases performed analyzing the Orphanet database estimated that between 263 and 446 million persons can be affected by a rare disease worldwide (5).

In the last 20 years, special attention has been dedicated to the “ultra-rare” diseases. This subset of RDs was informally introduced in 2004 by the UK National Institute for Health and Care Excellence (NICE), indicating diseases with a prevalence of <1 per 50,000 persons (6). This subset of diseases has been identified mainly due to the peculiar implications of developing specific orphan drugs. Richter and coworkers have delineated that orphan drugs designed for ultra-rare diseases form a unique subset, typically associated with elevated costs, less rigorous approval processes, and a greater likelihood of being biologic agents than conventional medications (7). Ultra-rare diseases present significant challenges to healthcare systems, primarily due to a chronic lack of diagnosis and a need for innovative treatment approaches. The prevalence of such diseases is rising sharply as genomic sequencing becomes more widespread, revealing a growing number of affected individuals (8).

Ultra-rare diseases can also fall into the category of diseases defined by the degree of research attention they receive, with many such conditions being considered neglected or under-researched since they experience a disproportionally low level of scientific study and medical scrutiny (9).

Finally, it is necessary to consider diseases lacking a specific diagnosis, the so-called “*undiagnosed rare diseases*” (URDs). Even in this instance, we lack a specific and shared definition of what should be considered an undiagnosed disease. Traditionally, rare conditions have been described with specific clinical features and, in some cases, named after their discoverer. Nonetheless, there are numerous cases where the precise cause of a medical condition remains elusive (10). Determining a practical course of treatment becomes challenging without a definitive etiological diagnosis. This uncertainty in pinpointing the exact origin of the disease applies not only to acquired diseases but also to those stemming from genetic factors. An accurate determination of the disease’s etiology is crucial; without it, healthcare providers often cannot tailor therapies that directly target the underlying problem, instead resorting to symptomatic management. In some cases, people affected by RDs, with a definite diagnosis, clear etiology, and specific treatments, remain undiscovered with substantial physical, psychological, and social consequences (11–14). Often, these persons can reach a diagnosis if evaluated at a reference Center with a high degree of specialization. In summary, URDs include pathological conditions without a name, diseases with a well-characterized phenotype but without a clear comprehension of the pathogenesis, and diseases with an unknown molecular basis or due to non-genetic factors, including the influence of environmental factors.

For these specific conditions, the complexity of the disease determines that a high percentage of cases must be referred to clinical centers with a high level of specialization and, finally, to undiagnosed RD programs (where they exist). These programs have developed systematic procedures based on an individualized and exhaustive study of patients by a multidisciplinary team.

In 2008, the National Institutes of Health (NIH) launched a study program dedicated to URDs (15). Since then, there have been over 1,000 papers indexed on PubMed concerning undiagnosed diseases. Furthering this effort, 2014 saw the creation of the Undiagnosed Diseases Network International (UDNI) Consortium, which currently includes nearly 180 centers worldwide (10).

Biomedical Centers with the necessary specialization and access to advanced laboratory, instrumental, and genetic analysis tools often need to be improved, particularly in low- and lower-middle-income countries. This scarcity makes it more challenging for people living with rare and undiagnosed diseases to obtain a diagnosis and treatment. The deficit in specialized facilities capable of conducting intricate testing often leads to significant roadblocks in the diagnostic journey for these individuals, compounding the hardships they face in managing their health conditions (16).

While acute undiagnosed infectious diseases rightfully command substantial public health attention due to their potential for rapid spread, chronic undiagnosed diseases, particularly those with a genetic basis, represent a persistent and often neglected public health challenge. Individuals with chronic undiagnosed diseases frequently undergo a prolonged diagnostic odyssey, resulting in significant disability, psychological distress, and social isolation. The impact goes beyond the individual, affecting families and burdening healthcare systems. This study concentrates on chronic undiagnosed diseases with a genetic etiology, as advancements in genetic testing and research provide promising opportunities for improving diagnosis and potentially leading to targeted treatments.

The present paper aims to analyze the results of a survey conducted among members of the UDNI consortium and external participants. The survey aimed to identify four main aspects related to undiagnosed diseases: (1) standard definitions for these conditions; (2) the availability of facilities and networks that respondents have access to at national and international levels; (3) the impact of Advocacy Groups in helping individuals with rare and undiagnosed diseases; and (4) potential initiatives to improve the patient journey toward diagnosis and treatment. By consolidating this information, we aim to shed light on the current situation and suggest ways to enhance the care of people living with URDs.

## Methods

This study adhered to the COnsolidated criteria for REporting Qualitative research (COREQ) guidelines, utilizing the 32-item checklist for interviews and focus groups (17).

### Data collection procedure

We initiated data collection on July 1, 2023, utilizing an online questionnaire created and managed through Microsoft Forms. The target demographic for this questionnaire included all 161 members of the UDNI at the time of the study. Members were invited by email to take part in the questionnaire voluntarily. We leveraged the UDNI consortium’s outreach by promoting the questionnaire through targeted emails and sharing the survey link on the official UDNI website.^1^ The initial September 10, 2023, deadline was extended to October 16, 2023, to increase participation. Furthermore, an additional email reminder was sent at the start of October 2023.

Due to voluntary participation in the online survey, the issue of non-participants is not applicable.

Following the survey closure on October 16, 2023, data were securely extracted from Microsoft Forms as an xlsx file.

### Questionnaire design and anonymity

The online survey was designed to be accessible to all respondents and to ensure complete anonymity to encourage honest and uninhibited responses. At no point were identifying details collected, ensuring that personal data could not be traced back to respondents. The questionnaire comprised a series of structured questions aimed to gather information on practices, challenges, and outcomes associated with the diagnosis within the global undiagnosed diseases community. The items also included free text fields, allowing for a comprehensive analysis of the diverse experiences and perspectives within the UDNI membership. Before the launch, the survey tool was pilot tested with a select group of UDNI members to ensure clarity, relevance, and ease of use. The items included in the questionnaire are accessible as supplementary material through the online version of this article. After closing the questionnaire, the data were extracted from Microsoft Forms for analysis, ensuring that the information remained confidential and secure throughout the study proceedings.

### Data analysis

Descriptive analysis was performed using Excel vs. 16.78 for Mac. Frequency analysis was performed using pivot tables.

Six authors (SB, SS, DT, MS, CC, and LC) performed a systematic qualitative analysis to elucidate prevalent themes and keywords. The methodology encompassed several distinct stages:

1. Data collection: the initial stage involved the aggregation of textual data from the free text answers derived from the survey (questions n…).
2. An initial coding framework was developed deductively based on the research questions and then refined inductively through an iterative process of reviewing and discussing the data. Themes were identified through constant comparison and discussion among the coders, ensuring consensus on the interpretation of the data.
3. Text Preprocessing: the raw text data underwent a preprocessing phase to standardize and clean the dataset. This process included the removal of all forms of punctuation and non-alphanumeric characters. Additionally, the text was converted to a uniform lowercase format to maintain consistency. A crucial aspect of this phase was eliminating commonly occurring words, known as “stop words,” which do not contribute significantly to the semantic analysis (and, is, or).
4. Word Frequency Analysis: following preprocessing, a quantitative analysis was conducted to ascertain the frequency of each unique word in the corpus. This involved the creation of a frequency distribution, wherein each distinct word was mapped to its corresponding occurrence count within the dataset. Data obtained from this phase were also summarized in tables.

To increase the trustworthiness of our qualitative analysis, we used several strategies, such as regular discussions with the entire research team to challenge assumptions and ensure diverse perspectives were included during the study. Additionally, we compared findings from open-ended survey responses with existing literature to confirm our interpretations.

All qualitative data were securely stored on an encrypted server to maintain confidentiality and facilitate future research.

## Results

### Respondents’ analysis

#### Respondent demographics and UDNI membership status

The survey successfully reached participants from 45 countries, with 90 individual answers. 41 countries belong to the UDNI. The survey also garnered responses from four countries not formally associated with the UDNI.

#### Geographical and continental distribution of respondents

Table 1A indicates the distribution of the respondents by continent; they were distributed across six continents with varying participation levels. Africa’s participation from seven countries totaling seven respondents demonstrates a one-to-one country-to-respondent ratio. The Americas reported participation from seven countries, contributing to a collective of 26 respondents, with a vast majority from the US. Asia was represented by 14 countries, with 21 individuals responding. Two countries bridging the continents of Asia and Europe (Georgia and Türkiye) accounted for seven participants. Europe saw the highest number of respondents, with 14 countries and a combined total of 27 respondents. Oceania, with one country participating, had two respondents.

**Table 1:** The table indicates the number of responding countries and individuals classified by continent, income category (according to the World Bank classification), the leading healthcare system (public national healthcare system, universal public or private insurance, mixed public and private insurance or non-universal healthcare system) and the healthcare coverage (according to the WHO classification).

(A)		
Continent	# of countries (%)	# of respondents (%)
Africa	7 (16)	7 (8)
America	7 (16)	26 (29)
Asia	14 (31)	21 (23)
Asia/Europe	2 (4)	7 (8)
Europe	14 (31)	27 (30)
Oceania	1 (2)	2 (2)
Total	45	90

(B)		
Income category	# of countries (%)	# of respondents (%)
High	15 (33)	37 (41)
Low	10 (22)	15 (17)
Medium	6 (13)	15 (17)
Medium high	3 (7)	3 (3)
Medium low	11 (24)	20 (22)
Total	45	90

(C)		
Healthcare system	# of countries (%)	# of respondents (%)
Non-universal insurance system	6 (13)	19 (21)
Universal government-funded health system	18 (40)	33 (37)
Universal private health insurance system	2 (5)	2 (2)
Universal public insurance system	11 (24)	17 (19)
Universal public-private insurance system	6 (13)	17 (19)
Unknown	2 (5)	2 (2)
Total	45	90

(D)		
Healthcare coverage according to WHO	# of countries (%)	# of respondents (%)
Limited coverage	10 (22)	25 (28)
Near universal health coverage	8 (18)	14 (16)
Partially covered system	9 (20)	14 (16)
Universal health coverage	18 (40)	37 (41)
Total	45	90

#### Respondents by income category and healthcare systems

Analysis of respondents according to the World Bank’s income classifications, illustrated by Table 1B, shows that high-income countries had 15 representatives, accounting for the largest group of respondents (37 individuals). Low-income nations, represented by 10 countries, had 15 respondents, highlighting the engagement across economic strata. Medium income was divided into three subcategories: 6 medium-income countries contributed 15 respondents; 3 countries were classified as medium-high, offering three respondents; and 11 countries fell into the medium-low category, providing 20 respondents.

The healthcare systems of the different countries were varied, with 33 respondents belonging to countries with universal government-funded health systems. A total of 11 countries and 17 respondents represented universal public insurance systems. Six nations had non-universal insurance systems, equating to 19 respondents. Additionally, universal health coverage systems with a mix of public and private insurance and those with solely private insurance saw representation from 6 and 2 countries, respectively, yielding 17 and 2 respondents.

#### Healthcare coverage insight

In terms of healthcare coverage, as classified by the World Health Organization (WHO), the survey captures a range of health service provision statuses (Table 1D). Eighteen countries, representing 37 respondents, reported having universal health coverage. Conversely, 10 countries with limited coverage resulted in 25 respondents demonstrating potentially significant barriers to healthcare access within this group. Near-universal coverage was recorded from eight countries with 14 corresponding respondents, indicating a substantial extent of healthcare inclusion. Nine countries fell under the partially covered system category, similarly accounting for 14 respondents. These insights into healthcare coverage are instrumental in understanding how the various healthcare models intersect with the challenges faced in diagnosing and managing undiagnosed diseases.

### Rare and undiagnosed diseases definitions

#### Which of the following definitions fit with your undiagnosed rare diseases activities?

**Figure 1:**
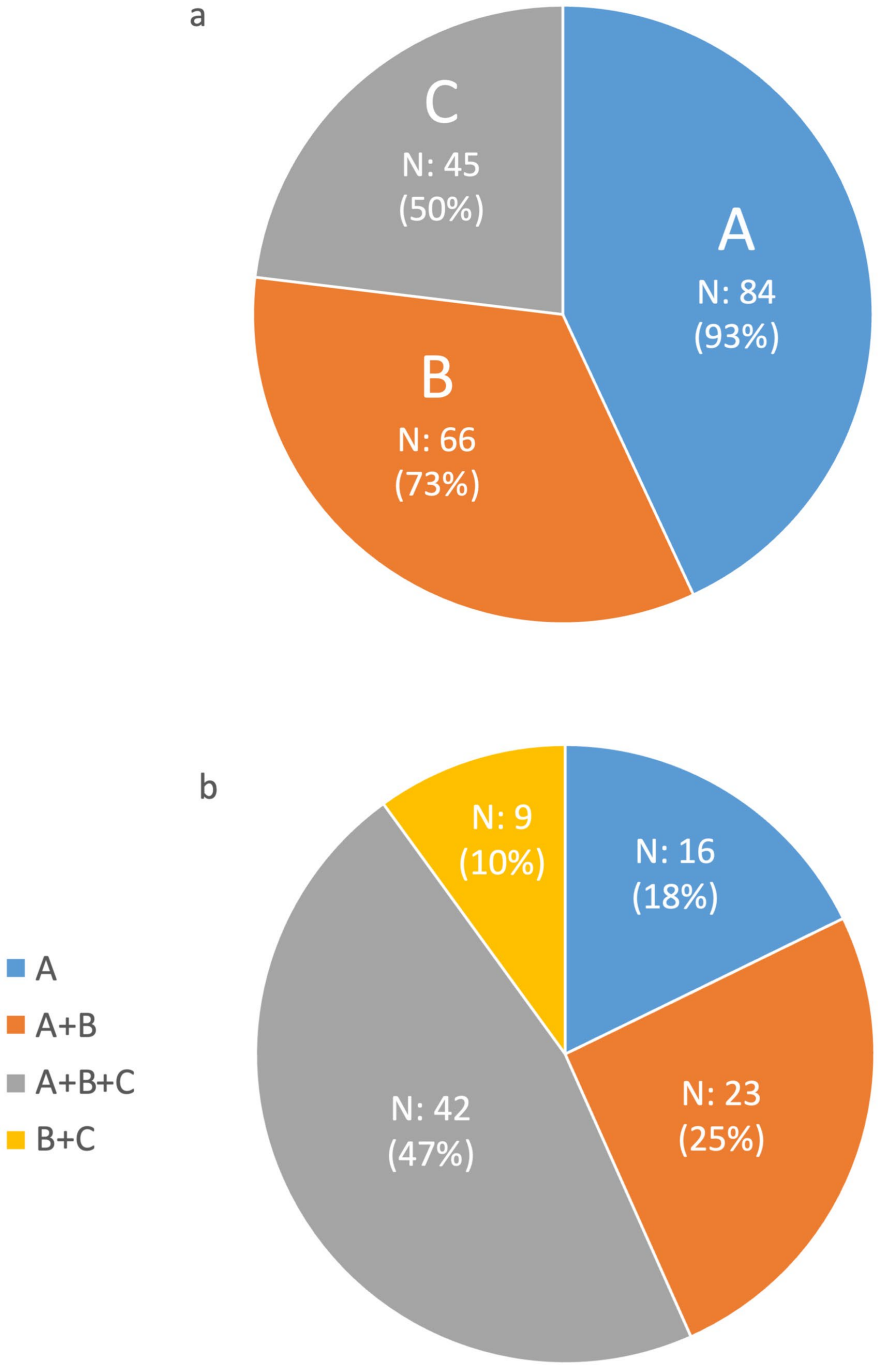
Distribution of the answer to the question “*Which of the following definitions fit with your undiagnosed rare diseases activities?*” The possible answers were (A) “*activities for people living with rare diseases that do not have a diagnosis yet*,” (B) “*activities for people living with rare diseases not yet discovered by medical science*,” and (C) “*activities related to misdiagnosis*.” The respondents could give multiple answers. **(A)** Illustrates the individual responses to the question; **(B)** considers the possible multiple combinations of responses.

The survey posed a critical question to participants to understand their perspectives on the nature of activities related to undiagnosed rare diseases. Specifically, the question was “*Which of the following definitions fit with your undiagnosed rare diseases activities?*” with three potential answers provided: (a) *“activities for people living with rare diseases that do not have a diagnosis yet”*; (b) “*activities for people living with diseases not yet discovered by medical science*”; and (c) “*activities related to misdiagnosis.”* This question permitted respondents to select multiple answers reflecting the multifaceted nature of their work. As illustrated in Figure 1A, 93.3% of participants (*N* = 84 of 90) answered with option (a), suggesting a consensus that a foundational aspect of undiagnosed rare disease activities involves working with people who are yet to receive a formal diagnosis.

A significant portion of the respondents, 73.3% (*N* = 66 out of 90), also agreed that their work encompasses activities for people suffering from diseases not yet recognized in the medical literature, as indicated by option (b). This reflects a considerable dedication to advancing medical knowledge and pursuing discovery in rare diseases within the community of clinicians and researchers dedicated to undiagnosed diseases. Additionally, more than half of the respondents (*N* = 50 out of 90) acknowledged engaging in activities to rectify misdiagnosis, highlighting the importance of reviewing and correcting diagnostic errors as a component of their endeavors within undiagnosed diseases.

Figure 1B shows that almost half of the survey’s respondents (46.7%) consider their contributions to span all three definitions provided (a, b, and c), indicating a comprehensive involvement in the broader spectrum of undiagnosed rare disease activities. This implies that most participants do not limit their scope to a single facet of rare disease work. Instead, they engage in a more integrated approach that includes assisting people without a diagnosis, exploring unknown medical phenomena, and addressing misdiagnoses. This complex scope of work underscores the intricate and comprehensive approaches employed by professionals when dealing with the challenges of undiagnosed diseases.

#### Consensus on the definition of “undiagnosed rare disease”

**Figure 2:**
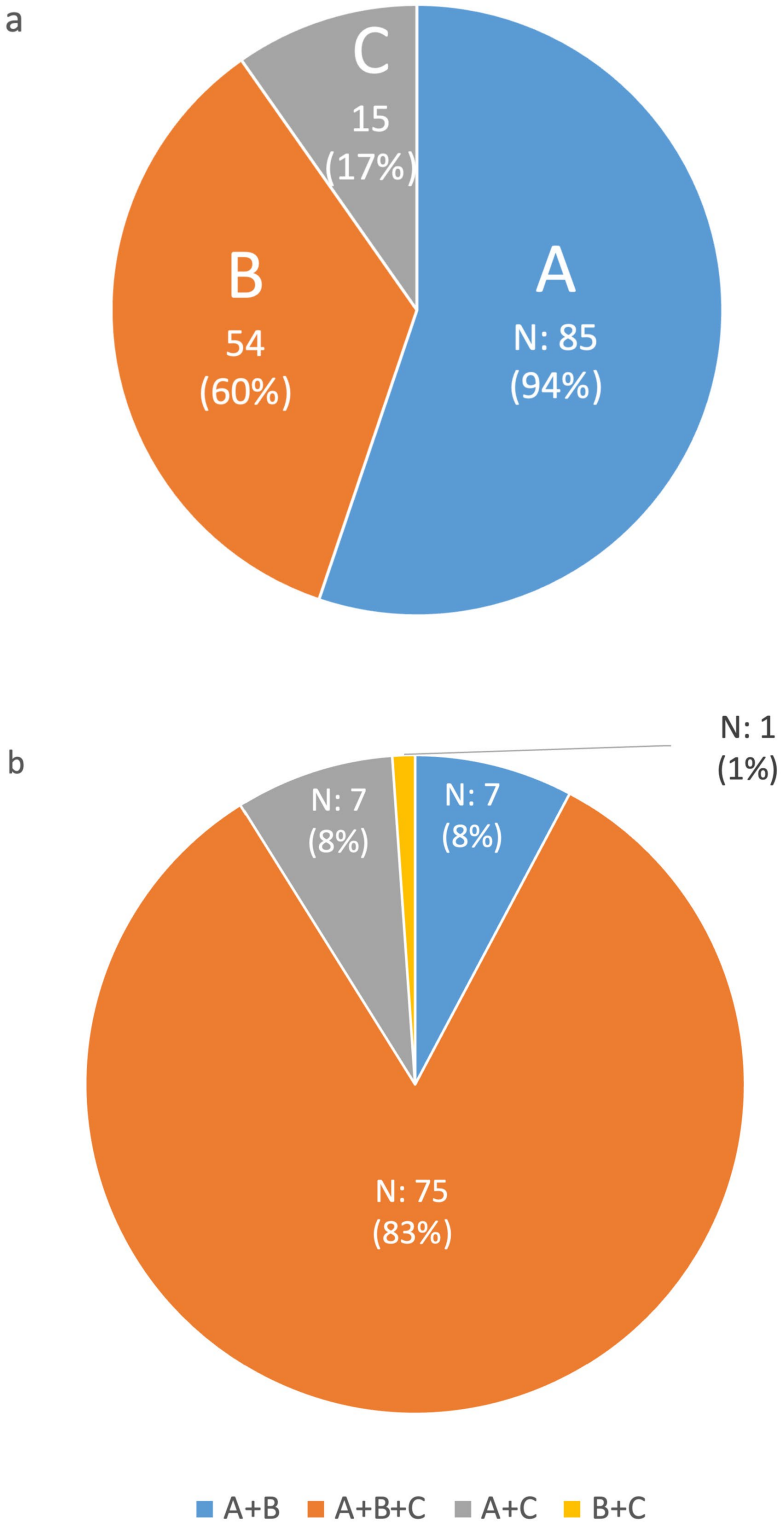
Distribution of the answer to the question “*Which of the following options should be included in the definition of ‘undiagnosed rare disease’ in your opinion?*” The possible answers were (A) “*People with unique characteristics of their disorder who have already been extensively examined, and for which obvious diagnoses have been discarded (UDNI definition)*,” (B) “*A new disease*,” and (C) “*Rare diseases missed diagnosis*;” the respondents could give multiple answers. **(A)** Illustrates the individual responses to the questions; **(B)** considers the possible multiple combinations of responses.

Participants were also queried on their opinions regarding the most accurate definition of “undiagnosed rare disease.” The question offered three potential descriptors, and respondents could endorse any combination. A remarkable 94.4% of participants concurred that the most comprehensive definition of an undiagnosed rare disease aligns with the UDNI description (Figure 2A). This definition characterizes undiagnosed rare diseases as conditions in which patient’s exhibit unique characteristics within their disorder, have undergone extensive evaluations, and all straightforward diagnoses have been systematically ruled out, as referenced in UDNI’s white paper (10). In addition to advocating for this definition, a significant majority, 83.3% of respondents, expressed that a complete definition should encompass all proposed elements: the extensive examination of patients (as per the UDNI’s definition), the concept of the disease being novel, and instances where rare diseases have been misdiagnosed (Figure 2B). These findings highlight a broad agreement among experts on adopting a multifaceted approach to defining undiagnosed rare diseases, underscoring the complexity and intricacy involved in diagnosing these elusive conditions.

## Availability of networks, infrastructures, and facilities for undiagnosed disease diagnosis and treatment

### National programs and strategies for undiagnosed rare diseases

**Figure 3:**
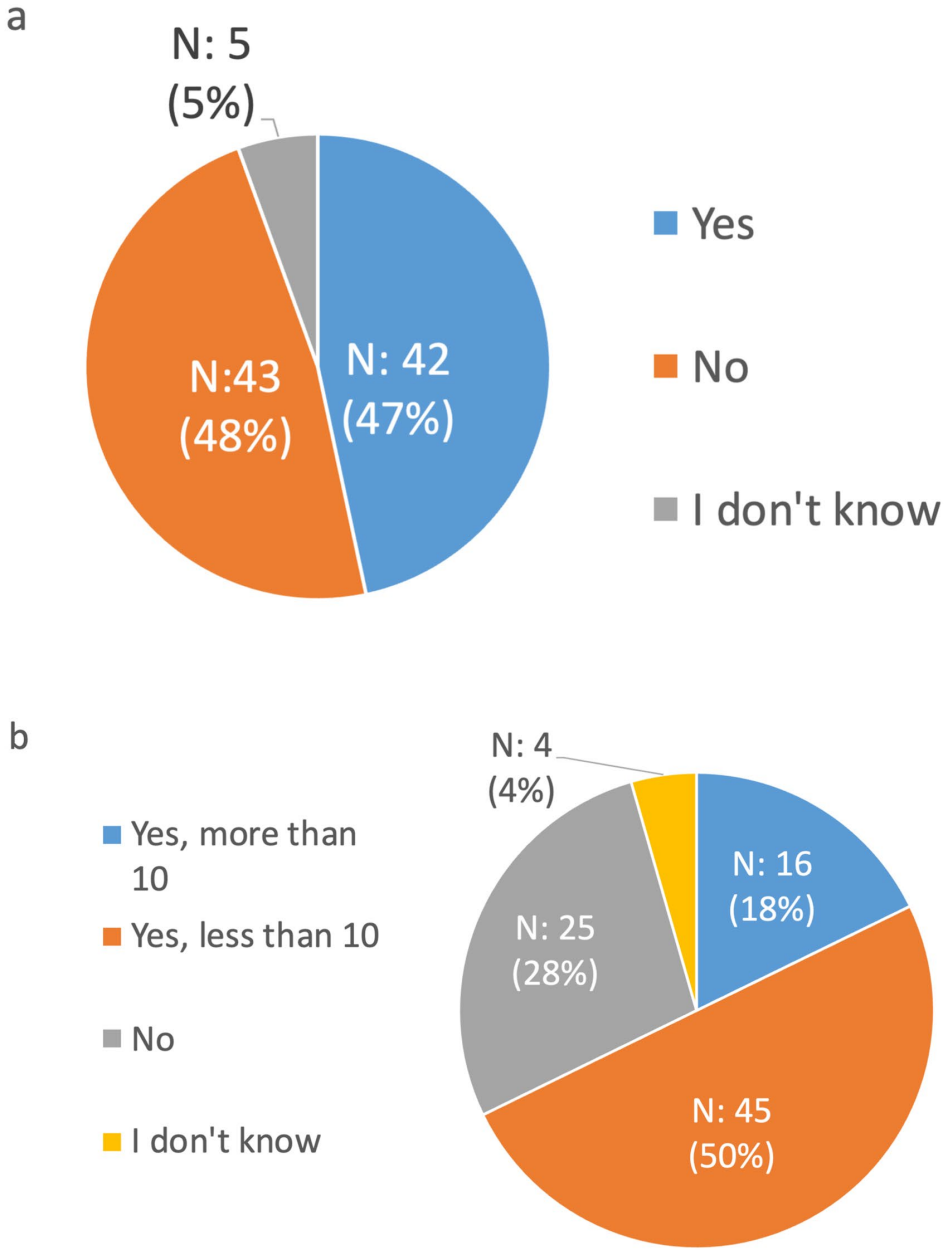
**(A)** Distribution of the answer to the question “*Is there a national program/strategy for undiagnosed rare diseases in your country?*” **(B)** Distribution of the answer to the question, “Are there centers dedicated to the care of undiagnosed rare diseases in your country?”

The survey posed two critical questions to participants to capture a snapshot of the infrastructure and policies for undiagnosed rare diseases. The first question queried whether there is a national program or strategy for undiagnosed rare diseases in the respondents’ respective countries, offering three responses: “Yes,” “No,” and “I do not know.” The second question sought to determine the existence and abundance of specialized centers dedicated to the care of undiagnosed rare diseases, with answer options including: “Yes, more than 10,”; “Yes, <10,”; “No,” and “I do not know.” As illustrated in Figure 3A, the survey revealed an equal split among participants acknowledging or denying the existence of national programs or strategies, signifying a notable divergence between countries regarding their systematic approach to these diseases. Strikingly, half of the respondents indicated that their countries host fewer than 10 such specialized centers, underscoring either a nascent development of dedicated care facilities or a modest scale of established centers, raising questions about the accessibility and adequacy of specialized care for undiagnosed rare disease persons (Figure 3B). This difference in responses articulates the contrasting landscapes in which patients and healthcare providers operate, from the macro level of national policymaking to the micro level of clinical care.

Healthcare professionals and researchers often seek alternative collaborative platforms requiring formalized national networks to tackle undiagnosed diseases. The survey elucidates this scenario, revealing that most respondents actively engage in existing networks despite a systemic void. According to the questionnaire data, a significant 78.9% affirmed their membership in national networks or organizations specializing in rare diseases, indicating a proactive approach within the respondent community to mitigate the resource gap for undiagnosed conditions. This engagement within national networks facilitates resource sharing and expertise exchange and underscores healthcare professionals’ commitment to navigating the complexities of rare disease diagnostics and management.

Taking networking a step further, the survey also probed the participants’ involvement in international consortia. 84.4% of participants confirmed their affiliation with international networks or organizations. Respondents’ propensity to engage internationally with the global community’s collective efforts against the challenges of rare and undiagnosed diseases. It highlights the importance of transnational cooperation in fostering advances in research, sharing crucial insights, and ultimately improving patient outcomes in a field where individual nations may lack the requisite infrastructure or initiative. The 15.7% who do not partake in such alliances may represent areas of potential growth for international collaboration, as outlined in the survey results.

#### Utilization and reimbursement of WES and WGS in different healthcare systems

**Figure 4:**
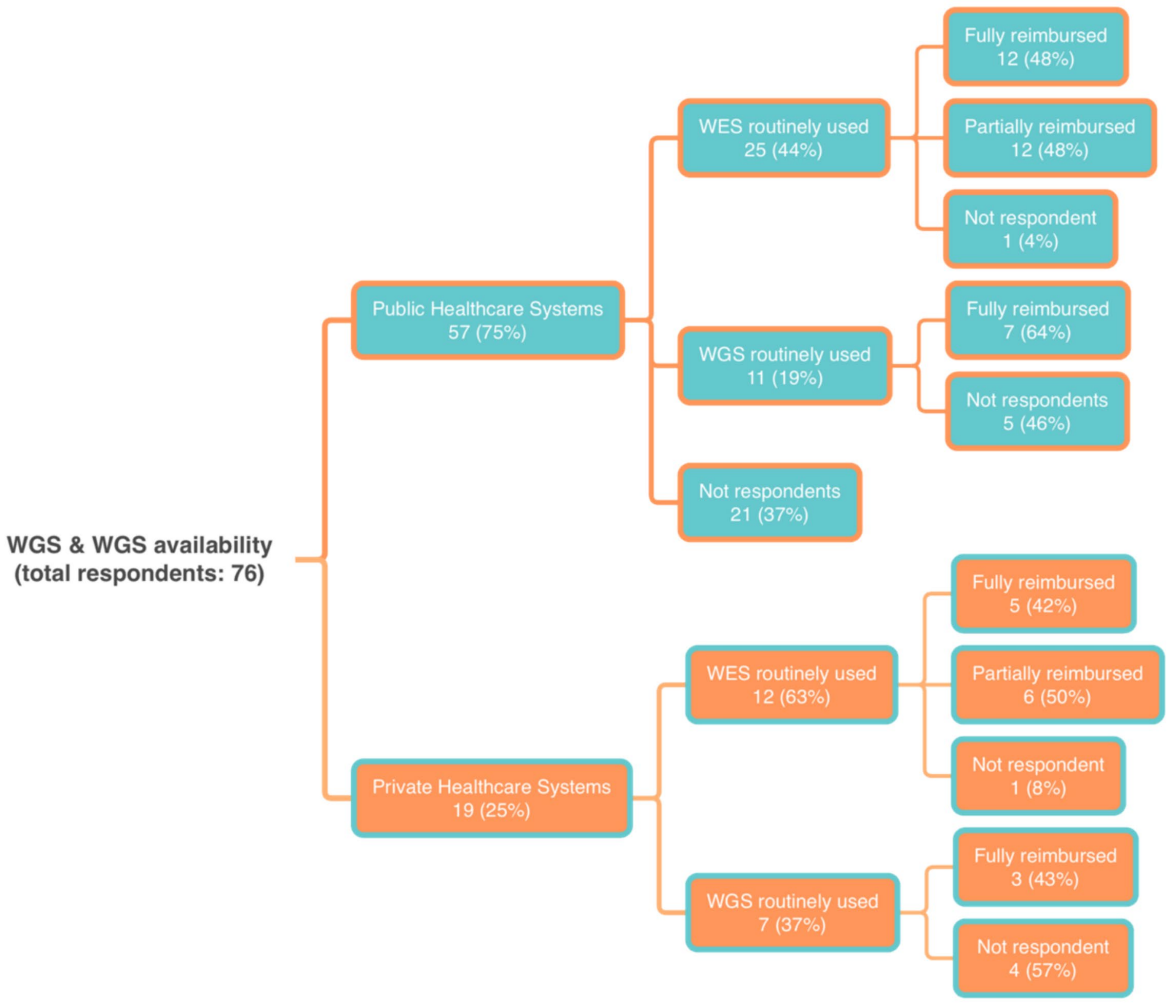
Distribution of the answers to the questions about the availability of Whole Exome Sequencing (WES) and Whole Genome Sequencing (WGS) within different healthcare systems.

We also examined the use and reimbursement of Whole Exome Sequencing (WES) and Whole Genome Sequencing (WGS) across different healthcare systems. We found that 63% of respondents (*n* = 57) were from countries with public healthcare. In these systems, WES was routinely used by 44% of respondents (*n* = 25), with 48% (*n* = 12) receiving full reimbursement (Figure 4). WGS has been less frequently reported as part of the standard of care, with only 19% (*n* = 11) of routine use. A large proportion (64%; *n* = 7) reported WGS reimbursement.

In private healthcare systems (21% of respondents), WES was routinely used by 63% (*n* = 12) and WGS by 37% (*n* = 7). For WES, 42% of respondents reported a complete reimbursement, while half reported only partial coverage. For WGS, 43% (*n* = 3) reported reimbursement.

## Role of advocacy groups

The survey incorporated a section to examine Advocacy Groups’ role and interactions with established networks for undiagnosed diseases. Of the respondents, 47.2% indicated an existing integration level of Advocacy Groups within their network.

A further analysis adopted “content analysis” to distill responses to 2 open-ended questions: (1) “*How are Advocacy Groups integrated into the management of undiagnosed rare diseases in your country?*”; and (2) “*In your opinion, how could Advocacy Groups be better integrated into managing undiagnosed rare diseases in your country?*” This process involved collecting descriptors by grouping words with similar meanings and assigning weights based on frequency. These synonyms were then categorized into descriptor categories and further classified into either “*opportunities*” or “*obstacles*” and “*Possible implementation,”* in line with the two questions. Variations in words were carefully examined for similar terms, spelling differences, and synonyms. Following a consensus among the coders, terms that exhibited word variations were considered synonymous (For example, “Education programs” and “education activities” were treated as synonymous.)

Tables 2, 3 concisely summarize the main findings. Descriptors were also used to generate a word cloud grouping words by synonyms, weighting them by frequency as previously described (data not shown).

**Table 2:** Analysis of challenges and opportunities of interactions with advocacy groups as emerged from the wording analysis of the responses to the questionnaire.

Opportunities	Co-design and integration at an executive level Interaction with advocacy groups and institutions of reference and discussion groups Co-development of education programs Facilitation of people living with URDs’ referral
Challenges	Complex integrations between clinical experts and advocacy groups

**Table 3:** Analysis of possible interactions and integrations with advocacy groups as emerged from the wording analysis of the responses to the questionnaire.

Research	Co-design of research studies and outcomes Development of registries Data sharing Participation in research studies
Education	Co-development of education programs for not experts in URDs (e.g., general practitioners, pediatricians) Co-development of education programs for people living with URDs and relatives
Policy making	Development of initiatives aiming to improve the awareness about URDs among stakeholders and citizen Co-development of national and international networks People living with URDs representation and inclusion in management committees

## Discussion

The answers to our survey about undiagnosed diseases came mainly from people from the countries directly participating in the UDNI. Although the request was initially sent to UDNI participants and made available on the UDNI website, the response rate, which is nearly 50% of the subjects contacted (86 out of 161), implies a strong representation and engagement from within the UDNI community, reflecting the network’s commitment to addressing the challenges associated with undiagnosed diseases. In addition to UDNI members, the survey garnered responses from a few countries not formally associated with the UDNI. This extended outreach suggests a broader interest and potential for collaboration in addressing undiagnosed diseases beyond the current UDNI framework.

### Definitional consensus and systemic disparities

The survey’s findings reinforce the necessity of an internationally recognized definition for undiagnosed rare diseases (URDs). The overwhelming support for the UDNI’s definition is a testament to the need for a universal lexicon that transcends the boundaries of different healthcare systems. Despite this consensus, the survey also unveils significant disparities based on the categorization of healthcare systems, as well as the economic status of countries. While high-income countries with comprehensive public healthcare systems seem better equipped to adhere to UDNI standards and provide care for people with URDs, those from private or non-universal healthcare systems, and particularly low to medium-income countries, face additional hurdles attributable to limited resources and inadequate healthcare infrastructure. This emphasizes the crucial role of economic and healthcare policy in the equitable diagnosis and treatment of URDs.

The estimated prevalence of RDs globally suggests that a significant portion of the population could be affected, but the diagnostic odds remain unevenly distributed. Healthcare providers in high-income countries are more likely to have access to advanced diagnostic tools such as WES and WGS, which are often reimbursed by their healthcare systems. In contrast, such resources are scarce and rarely subsidized in lower-income regions. This discrepancy illustrates an economic divide and reflects on the efficacy of the different healthcare models in addressing complex, undiagnosed conditions. The insight gained underscores an urgent need for more accessible and affordable healthcare for all, regardless of geographic location or economic status.

Our survey revealed a potential knowledge gap regarding national programs for undiagnosed rare diseases. While five respondents were uncertain about the existence of such programs (Figure 1), fewer were unsure about dedicated care centers (Figure 2). This suggests that some respondents might be aware of care centers without overarching national strategies. This result could be due to decentralized care models, targeted awareness campaigns, or limitations in survey design. However, the anonymity of respondents prevents further exploration of this discrepancy, highlighting the trade-off between anonymity and detailed analysis in survey research.

### Tailoring strategies across diverse economic landscapes

The survey also draws attention to the disparate options available to people living with URDs, which vary significantly across income categories and healthcare systems. Furthermore, the underutilization and limited reimbursement for WGS in public healthcare settings suggest that even in developed nations, hurdles persist in integrating cutting-edge science into standard practice. Bridging these disparities is essential for achieving equitable health outcomes and entails fostering international collaboration focused on developing and disseminating advanced medical approaches (18).

### The importance of education and information

The analysis of responses reveals that most interviewees feel that healthcare workers (both medical and non-medical) require more training on undiagnosed diseases. They also suggest that patients, their families, and the public need more information and education. Interestingly, in this last area, one participant noted the animistic and supernatural perceptions of the causes of rare and undiagnosed diseases in some populations. In these cases, close collaboration between international networks such as UDNI and local operators is essential to increase awareness of the causes of diseases among the population, reduce stigma, and allow for better diagnosis and treatment of people living with URDs.

## Conclusion

While this study offers valuable insights into the challenges and opportunities surrounding undiagnosed diseases, it is important to acknowledge certain limitations.

The primary focus on UDNI member countries might not fully capture the experiences of individuals and healthcare systems in non-member countries. Additionally, the reliance on self-reported data could introduce potential biases. Further research employing diverse data collection methods and involving a broader range of participants is needed to confirm and extend these findings.

Nonetheless, the results of this survey show how crucial it is to mitigate the stark disparities among different countries. The implications of these findings extend beyond the UDNI network. A universally recognized definition for URDs could facilitate international collaboration in research, data sharing, and developing standardized diagnostic and treatment protocols. Addressing the systemic disparities identified in this study is crucial for ensuring equitable access to care and improving the lives of individuals with URDs worldwide.

While most respondents are part of national or international networks, fostering a more substantial engagement and resource-sharing ethos among member countries is critical. High-income countries could contribute by aiding lower-income counterparts through technology transfer, training, and research collaborations, enhancing diagnostic capabilities and treatment accessibility. To this end, a unified commitment to prioritizing URDs on the global health agenda, paired with targeted funding, stipulated national strategies, and aligned international cooperation, is imperative to leveling the playing field for the diagnosis and management of URDs, and capitalizing on the potential of advocacy groups as allies in this endeavor. Following its 10-year activity, the added value of UDNI is apparent. Networking has fostered several new activities, and the UDNI’s role in describing disparities and inconsistencies of approaches among countries has become evident (19). Based on the outcomes of the first decade, UDNI partners can act as ambassadors to strengthen the global effort to make undiagnosed RD more recognizable, thus paving the way to better healthcare.

Future studies should compare healthcare systems and income levels to understand URDs diagnosis and care globally. Longitudinal studies could track policy intervention impacts and the adoption of a universal definition of outcomes. Qualitative research on people living with URDs would offer insights into psychosocial effects and inform patient-centered care models.

This study aligns with health equity frameworks emphasizing the need to address social determinants for equitable outcomes. Systemic disparities in URDs diagnosis and care reflect broader healthcare inequalities. Future interventions should consider these determinants to create a just healthcare system for all, regardless of diagnosis or socioeconomic background.

Despite many efforts to solve the unsolved and to share experiences and protocols, a standard view and definition of rare and undiagnosed that incorporates the fast-evolving diagnostic landscape is needed (20).

## Data Availability

Raw data, consisting in the survey administered to UDNI members participating to the present initiative, are available from the authors, without undue reservation.

## Footnotes

^1^ https://www.udninternational.org
